# Physicochemical properties of calcium silicate-based formulations MTA Repair HP and MTA Vitalcem

**DOI:** 10.1590/1678-7757-2017-0115

**Published:** 2018-03-26

**Authors:** Bruno Martini Guimarães, Carlo Prati, Marco Antonio Hungaro Duarte, Clovis Monteiro Bramante, Maria Giovanna Gandolfi

**Affiliations:** 1 Università di Bologna, Dipartimento di Scienze Biomediche e Neuromotorie, Laboratorio di Biomateriali e Patologia Orale, Bologna, Italia; 2 Università di Bologna, Reparto di Endodonzia del Dipartimento di Scienze Odontostomatologiche, Bologna, Italia; 3 Universidade de São Paulo, Faculdade de Odontologia de Bauru, Departamento de Dentística, Endodontia e Materiais Odontológicos, Bauru, São Paulo, Brasil

**Keywords:** Physical properties, Materials testing, Endodontics, Calcium silicate, Dental materials

## Abstract

**Objective:**

This study aimed to analyze the following physicochemical properties: radiopacity, final setting time, calcium release, pH change, solubility, water sorption, porosity, surface morphology, and apatite-forming ability of two calcium silicate-based materials.

**Material and methods:**

We tested MTA Repair HP and MTA Vitalcem in comparison with conventional MTA, analyzing radiopacity and final setting time. Water absorption, interconnected pores and apparent porosity were measured after 24-h immersion in deionized water at 37°C. Calcium and pH were tested up to 28 d in deionized water. We analyzed data using two-way ANOVA with Student-Newman-Keuls tests (p<0.05). We performed morphological and chemical analyses of the material surfaces using ESEM/EDX after 28 d in HBSS.

**Results:**

MTA Repair HP showed similar radiopacity to that of conventional MTA. All materials showed a marked alkalinizing activity within 3 h, which continued for 28 d. MTA Repair HP showed the highest calcium release at 28 d (p<0.05). MTA Vitalcem showed statistically higher water sorption and solubility values (p<0.05). All materials showed the ability to nucleate calcium phosphate on their surface after 28 d in HBSS.

**Conclusions:**

MTA Repair HP and MTA Vitalcem had extended alkalinizing activity and calcium release that favored calcium phosphate nucleation. The presence of the plasticizer in MTA HP might increase its solubility and porosity. The radiopacifier calcium tungstate can be used to replace bismuth oxide.

## Introduction

Physicochemical properties of calcium silicate-based cements, such as ion release, solubility, porosity, setting time and radiopacity, are of the utmost importance as far as their clinical usefulness is concerned. Their good biological properties are attributed to their capacity for alkalinizing activity and calcium release^6^ ^,^ ^25^. In addition, the capacity to spontaneously produce a calcium phosphate apatite-like layer on their surface when in contact with phosphate-containing fluids is largely attributed to calcium release and maintenance of a high pH for a long period of time^13^ ^,^ ^14^ ^,^ ^25^.

Conventional mineral trioxide aggregate (MTA) cements are calcium silicate-based materials mainly composed of Portland cement, with the addition of bismuth oxide as a radiopacifier^5^. It has been shown that even small chemical differences^9^ ^,^ ^13^ or inclusion of additives in low percentages^1^ ^,^ ^2^ ^,^ ^6^ ^,^ ^8^ ^,^ ^10^ ^,^ ^28^ or even changes in the radiopacifying agent^2^ ^,^ ^25^ ^,^ ^27^ may strongly modify the physicochemical behavior of these materials.

Recently, new formulations have been introduced^9-11,27,28^. Among them, MTA Repair HP (Angelus, Londrina, PR, Brazil) and MTA Vitalcem have been proposed.

MTA Repair HP is based on the formulation of conventional MTA but contains calcium tungstate as radiopacifier and a mixing liquid with a plasticizer agent. It is proposed for use as root-end filling, pulp capping, pulpotomy, apexogenesis, apexification and to repair root canal perforations. According to the manufacturer's instructions, this new formula maintains the chemical properties of the original MTA, but improved its physical properties related to manipulation.

No further information is available and no studies have been published.

MTA Vitalcem cement has a composition similar to that of conventional MTA^7^ but contains zirconium dioxide as radiopacifier. MTA Vitalcem has been proposed as root-end filling, perforation repair, root resorption, apexification, and pulp capping. It has shown antimicrobial properties^24^ and regenerating properties^7^ similar to those of conventional MTA.

Calcium silicate-based cements seem to have intrinsic properties suitable for their clinical use such as good sealing, bioactivity, and good biological properties. Therefore, new calcium silicate MTA-like cements have recently been introduced. In the literature up to now, there are no studies on the physicochemical characterization of MTA Repair HP and MTA Vitalcem.

Thus, the aim of this study was to investigate the calcium release, pH, solubility, porosity, water sorption, radiopacity, and calcium phosphate nucleation in simulated body fluid of MTA Repair HP and MTA Vitalcem.

## Material and methods

### Material

**Figure 1:**
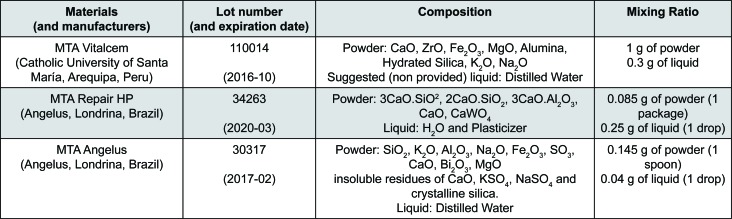
Composition of the materials used

The materials used in the study were the MTA Repair HP, MTA Vitalcem and White MTA Angelus (control) (Figure 1). We prepared the MTA Repair HP using 0.17 g of powder to 2 drops of liquid. We mixed the cement in a glass plate using a metallic spatula for 40 s to obtain a homogeneous consistency, as recommended by the manufacturer. Were prepared the MTA Vitalcem and the MTA Angelus using a 3:1 powder-to-liquid ratio. All materials were prepared according to the manufacturer's instructions.

### Physical properties: radiopacity, setting time, porosity, solubility and water sorption

We analyzed the radiopacity following the Gandolfi, et al.^10^ (2012) methodology, in accordance with ISO 6876:2002. Three samples were prepared for each cement. We radiographed freshly mixed samples (approximately 10 mm diameter; 1.0 mm height each) using a radiographic unit (Myray Cefla, Imola, Italy) with an aluminum step wedge (60 mm long, 10 mm wide) as reference. Afterwards, we evaluated the digitized radiographs and converted the radiographic density values into aluminum step-wedge equivalent thickness (mm Al) using ImageJ software (U.S. National Institutes of Health, Bethesda, Maryland, USA).

To establish the setting time (n = 3 for each material), according to the ASTM specification Number C266-08 guideline, we compacted the freshly mixed pastes into polyvinyl chloride molds (10 mm diameter, 2 mm thickness) and stored them at 37°C with 95±5% relative humidity. The final setting time was registered when no indentation was caused by a needle weighing 456.5 g, with a tip diameter of 1.06 mm.

For porosity, another set of disks (compacted into polyvinyl chloride molds) (8±0.1 mm diameter × 1.6±0.1 mm) (n = 10 for each material) were set at 37°C and 99% relative humidity for a period equal to 70% of their setting time^9^ ^,^ ^14^ in compliance with ISO 6876. We weighed demolded specimens to determine the initial mass (D_i_) and vertically immersed them in 20 mL of distilled water at 37°C. After 24 hours, the mass while suspended in water (S) was determined and (after removing the excess water from the surface of each sample with moistened filter paper) the saturated mass (M) was recorded^9^ ^,^ ^11^ ^,^ ^14^. Samples were dried at 37°C until the weight was stable [dry mass (D_f_)]. We repeated each weight measurement three times to the nearest 0.001 g using an analytical balance (Bel Engineering series M, Monza, Italy).

The solubility {S=[(I – D)/D]×100} and the water sorption {WS=[([M - D_f_])/D_f_]×100} were calculated as percentage of the original weight^9-11^. The exterior volume V (V=M - S), open pore volume V_OP_ (V_OP_=M - D), impervious portion volume V_IP_ (V_IP_=Df - S), and apparent porosity P {P=[([M - D_f_])/V]×100} were calculated by Archimedes' principle by following the Gandolfi, et al.^9^ ^,^ ^11^ ^,^ ^12^ (2015, 2014, 2015) method.

### Alkalinizing activity (pH of soaking water) and calcium release

The test was performed as described by Gandolfi, et al.^11^ (2014). Ten samples of each cement polyvinyl chloride mold (8±0.1 mm diameter × 1.6±0.1 mm, n=10 for each material) with the cements were immersed in 10 mL of deionized water at 37°C^9^ ^,^ ^11^ ^,^ ^14^. The soaking water was collected and replaced after time intervals of 3 and 24 h, and 7, 14, 28 d^9^ ^,^ ^11^. We analyzed calcium ions (ppm) and alkalinizing activity (pH) of the soaking water by placing the beaker containing it on a magnetic stirrer, and using a multiparameter laboratory meter (inoLab 750 WTW, Weilheim, Germany) connected to a calcium probe (Calcium electrode; Eutech instruments Pte Ldt, Singapore) or a (selective) temperature-compensated pH probe/electrode (Sen Tix Sur WTW, Weilheim, Germany). For the calcium analysis, we supplemented the solution with 200 μL (2%) ionic strength adjuster (4 mol/L KCl, WTW, Weilheim, Germany).

### Calcium phosphate nucleation

Freshly mixed samples (8±0.1 mm diameter × 1.6±0.1 mm) were vertically immersed in 20 mL of HBSS (Hank's Balanced Salt Solution, Lonza Walkersville, Inc., Walkersville, MD, USA) by following the Gandolfi, et al.^9^ ^,^ ^13^ (2015, 2013) method and stored at 37° for 28 d. The HBSS was renewed weekly. Fresh samples and 28-day-old samples were examined using an environmental scanning electron microscope (ESEM; Zeiss EVO 50, Carl Zeiss, Oberkochen, Germany) connected to an energy dispersive x-ray (EDX; Oxford Instruments, Abingdon, UK)^17^. EDX data were used to calculate the surface calcium-to-phosphorus (Ca/P) atomic ratio^9^ ^,^ ^11^ ^–^ ^13^.

### Statistical analysis

The normally distributed data were analyzed by parametric testing (Sigma Stat, San Jose, CA, USA) by using two-way ANOVA followed by the RM Student-Newman-Keuls test (statistically significant difference for p<0.05).

## Results

### Physical properties: radiopacity, setting time, porosity, solubility and water sorption

Radiopacity, final setting time, solubility, porosity and water sorption are summarized in Table 1.

**Table 1:** Radiopacity, final setting time, solubility, porosity and water sorption of the tested materials. Different small letters represent statistically significant differences (two-way ANOVA followed by Student-Newman-Keuls with p<0.05) in the same column

	Radiopacity (mm Al)	Final Setting Time (m)	Solubility (%)	Exterior Volume (cm^3^)	Volume of Open Pores (cm^3^)	Volume of Impervious Portion (cm^3^)	Apparent Porosity (Vop/V %)	Water sorption (%)
MTA Vitalcem	2.46±0.17^b^	140±2ᵃ	14.19±1.32ᵃ	0.0913±0.0056ᵃ	0.0286±0.0010^b^	0.0627±0.0049ᵃ	31.37±1.38ᵃ	18.14±1.07ᵃ
MTA Repair HP	4.50±0.46ᵃ	85±2.64^b^	8.18±1.74^b^	0.0877±0.0045ᵃ	0.0258±0.0006^b^	0.0619±0.0044ᵃ	29.45±1.49ᵃ	14.96±0.95^b^
MTA Angelus	5.81±0.55ᵃ	84.33±5.13^b^	4.91±3.73^c^	0.0873±0.0015ᵃ	0.0218±0.0033ᵃ	0.0655±0.0037ᵃ	25.02±3.89^b^	12.04±2.60^c^

MTA Repair HP showed similar radiopacity to that of conventional MTA, with no statistical difference (p>0.05), whereas MTA Vitalcem presented the lowest radiopacity.

MTA Repair HP showed similar final setting time values to those of conventional MTA, with no statistical difference (p>0.05). MTA Vitalcem presented markedly higher values that differed statistically from both those of MTA Repair HP and conventional MTA.

Conventional MTA showed the lowest values of solubility, open pore volume, apparent porosity and water sorption (p<0.05); while MTA Vitalcem had the highest values of water sorption and solubility (p<0.05). MTA Repair HP and MTA Vitalcem showed similar open pore volume and apparent porosity values. However, MTA Repair HP had significantly lower solubility and water sorption values when compared with MTA Vitalcem (p<0.05).

### Alkalinizing activity (pH of soaking water) and calcium release

The alkalinizing activity and calcium release values are summarized in Table 2 (sections A and B).

**Table 2:** (A) pH of soaking water; (B) Calcium ions released in soaking water. Different capital letters represent statistically significance differences (p<0.05) in the same line, whilst different small letters represent differences in the same column

(A) pH of soaking water	3 hours	1 day	3 days	7 days	14 days	28 days
MTA Vitalcem	11.54±0.09^Aa^	11.51±0.54^Aa^	11.15±0.30^Aa^	11.11±0.28^Aa^	9.13±0.60^Bb^	9.59±0.48^Ca^
MTA Repair HP	11.37±0.06^Aa^	11.30±0.09^Aa^	11.03±0.10^Aa^	11.24±0.47^Aa^	9.23±0.47^Bb^	9.05±0.35^Ba^
MTA Angelus	11.34±0.31^Aa^	11.28±0.60^Aa^	11.08±0.63^Aa^	11.20±0.44^Aa^	10.31±0.51^Ba^	9.13±0.49^Ca^
distilled water	6.7±0.3^Ab^	6.8±0.1^Ab^	7.2±0.4^Ab^	7.1±0.1^Ab^	6.5±0.6^Ac^	7.2±0.5^Ab^
**(B) Calcium released (ppm)**	**3 hours**	**1 day**	**3 days**	**7 days**	**14 days**	**28 days**
MTA Vitalcem	143.1±7.10^Aa^	45.11±6.35^Bb^	35.85±4.32^Ca^	24.79±3.90^Db^	7.58±1.18^Fc^	11.85±0.92^Eb^
MTA Repair HP	48.81±3.24^Bb^	60.77±4.80^Aa^	40.32±3.54^Ca^	51.04±4.83^Ba^	21.02±2.11^Da^	14.80±1.58^Ea^
MTA Angelus	49.65±6.97^Ab^	33.77±7.82^Bc^	23.91±6.08^Cb^	50.64±2.63^Aa^	17.47±4.87^Db^	11.53±1.25^Eb^
distilled water	1.0±0.8^Ac^	0.6±1.1^Ad^	1.8±0.7^Ac^	1.6±0.8^Ac^	1.2±0.9^Ad^	1.3±0.3^Ac^

All materials produced an elevated pH during the first 7 d. Subsequently, the decrease in their alkalinizing activity was statistically significant. At 14 d, conventional MTA produced a statistically significant higher pH whilst at 28 d the alkalinizing activity was similar for all materials, with no statistical difference (p>0.05).

The calcium release decreased with time for all materials. The calcium release after 3 h was markedly higher for MTA Vitalcem in comparison with conventional MTA and MTA Repair HP (p<0.05); however, from 1 d up to 28 d, the MTA Repair HP calcium release was the highest and statistically higher than that of both conventional MTA and MTA Vitalcem (p<0.05). The calcium release of MTA Repair HP was the most stable and considerable among materials. Calcium release diminished for all materials as from 14 d.

### Calcium phosphate nucleation

**Figure 2:**
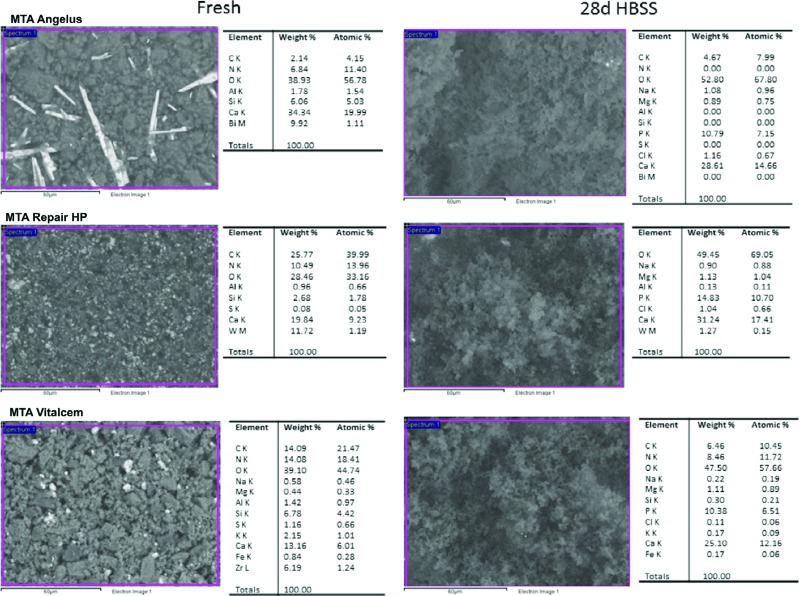
ESEM/EDX of freshly prepared and 28-day-old samples with the qualitative semiquantitative elemental composition of the materials

ESEM/EDX results of freshly prepared and 28-day-old samples with the qualitative semiquantitative elemental composition are shown in Figure 2.

The Ca/P deposits differing in amount and size were detected by ESEM on the surface of materials after 28 d immersion in HBSS.

Freshly mixed conventional MTA cement showed high calcium (Ca) (34.34% wt) and silicon (Si) (6.06% wt) content, and traces of aluminum (Al) (1.78% wt). Evident elongated particles of bismuth were visible. Following 28 d in HBSS the surface was coated with irregularly distributed Ca (28.61% wt) and P (10.79% wt) precipitates (Ca/P=2.05), and Bi was not detectable.

Freshly mixed MTA Repair HP showed a uniform surface containing interspersed granules of tungsten (W) (11.72% wt), and displayed Ca (19.84% wt) and Si (2.68% wt). After 28-d soaking in HBSS, the surface was covered with globular precipitates, the Si component disappeared and Na (0.90% wt), Mg (1.13% wt), and P (14.83% wt) elements from HBSS became detectable.

Freshly mixed MTA Vitalcem displayed a granular surface showing mainly Ca (13.16% wt) and Si (6.78% wt). Al (1.42% wt), Zr (6.19% wt) and S (1.16% wt), were noted. After 28 d in HBSS, the surface was coated with globular precipitates. The P (10.38% wt) element appeared, and amounts of Na (0.22% wt), K (0.17% wt) and Mg (1.11% wt) were also noted. Zr, Al and S components became undetectable.

## Discussion

In this study, two calcium silicate MTA based cements, each containing a different radiopacifying agent alternative to bismuth oxide, were compared with conventional MTA regarding their physical properties, ion release and ability to nucleate calcium phosphate on their surface.

Bismuth oxide has been hypothesized as being the component responsible for the color alteration of MTA and consequently tooth discoloration^22^ ^,^ ^29^ ^,^ ^30^. Reports on bismuth oxide interfering with MTA hydration and causing deterioration in the mechanical properties^5^ have encouraged research on its substitution by other radiopacifiers. Zirconium oxide and calcium tungstate are the radiopacifier of MTA Vitalcem and MTA Repair HP, respectively. Marciano, et al.^22^ (2014) revealed that none of these radiopacifiers induced cement discoloration in contact with collagen. Duarte, et al.^20^ (2009) showed that both radiopacifiers associated with Portland cement in a ratio of 20% met the ISO 6876 requirements (radiopacity 3.3 mm Al), displaying a radiopacity of 3.41 mm Al for the cement with zirconium oxide and 3.11 mm Al with calcium tungstate^20^. Another study comparing conventional MTA with these radiopacifiers associated with PC (in the same ratio as was proposed by the previous study) showed that all materials promoted calcium release and an alkaline pH, therefore, the authors considered calcium tungstate and zirconium oxide potential radiopacifying agents to be used in combination with PC^19^. In our results, MTA Repair HP and conventional MTA presented similar radiopacity values (5.81 and 4.50 mm Al, respectively) meeting the requirements of ISO 6876, while the radiopacity of MTA Vitalcem (2.46 mm Al) did not reach the recommended value, probably because it possess a radiopacifier ratio lower than 20%, thus compromising the radiographic visualization.

In endodontic surgery, cements with long setting times are more susceptible to washout and dissolution^14^. For this reason, laboratory studies assessing the setting times and the solubility of endodontic cements may be of interest to clinicians. In our study, the final setting time of conventional MTA was about 84 min, in agreement with previous studies^21^ ^,^ ^25^. MTA Repair HP showed a similar final setting time (85 min), without statistical differences when compared with conventional MTA. The setting time of MTA Vitalcem was the longest (140 min), with statistical differences; the longer setting time may make this cement more susceptible to dissolution.

The solubility of calcium silicate-based cements is related to the formation of soluble calcium salts and calcium hydroxide during the hydration and to the setting reactions of the material^9^. In this study both cements had solubility higher than the recommended by the ISO standards (3%). Conventional MTA showed a solubility of 4.91%, which is in agreement with a study that reported a solubility of 3.47%, even though a different methodology was used^29^. Another study that used the same methodology as ours displayed a higher solubility (29.55%)^15^. These differences could be correlated with changes in the formulation, as highlighted by the EDX data. MTA Repair HP showed a statistically higher solubility (8.18%) compared with that of conventional MTA. This difference between conventional MTA and MTA Repair HP, which have similar compositions, could be due to the plasticizer contained in the mixing liquid of MTA Repair HP. From our laboratory experience, the plasticizer (included to facilitate its manipulation and insertion into the root cavity, according to the manufacturer's brochure) seemed to improve the manipulation of MTA Repair HP compared with conventional MTA (4.91%). However, as a result, the material was more soluble. In addition, Cintra, et al.^4^ (2017) showed that MTA Repair HP has a good cellular viability, which could be related to the solubility of this material. The solubility of MTA Vitalcem (14.19%) was markedly higher than that of the other materials.

In this study, all materials showed alkalinizing activity and calcium release. These properties of calcium silicate cements are due to both the formation of calcium hydroxide and the release of calcium from the calcium silicate particles^17^ ^,^ ^25^. The materials presented similar alkalinizing activity results from 3 h to 7 d; statistically significant higher values were obtained for conventional MTA at 14 d. Conventional MTA showed similar results compared with those of other studies^13^ ^,^ ^26^ ^,^ ^31^. The decrease in alkalinizing activity with time, observed in our results, was also shown by Prati and Gandolfi^25^ (2015). Other studies have found lower values of pH in all periods^19^ ^,^ ^21^.

The release of calcium is a key factor for successful pulp capping therapy because of the action of calcium on pulp cell differentiation and hard tissue mineralization^9^ ^,^ ^18^ ^,^ ^23^ ^,^ ^25^. In our study, MTA Vitalcem showed the highest initial (3 h) calcium release (143.1 ppm), decreasing with time until 14 d, when it presented the lowest release (7.58 ppm) compared with all tested materials. MTA Repair HP showed the highest release (p<0.05) values from 1 d to 28 d. Conventional MTA showed the highest calcium release at 7 d (50.64 ppm), presenting similar results to those of other studies^9^ ^,^ ^25^. A recent study, with similar evaluation time intervals as ours, found lower calcium release values^21^.

MTA Vitalcem showed the highest water sorption value (18.14%); this result could be correlated with its highest values of calcium release, final setting time, and open and apparent porosity (31.37%). For MTA Repair HP, the high water sorption (14.96%) could be correlated to the presence of the plasticizing agent. Conventional MTA showed statistically significant lower values of water sorption (12.04%) and apparent porosity (25.02%).

The growth of a calcium phosphate (apatite) layer creates an ideal environment for stem cell and osteoblast differentiation and colonization to support new bone formation^9^ ^,^ ^14^ ^,^ ^25^. ESEM/EDX analysis provided qualitative and semiquantitative measurements of atomic calcium and phosphorous. The high intensity of peaks for Ca and P detected in EDX analysis was indicative of precipitation of amorphous deposits corresponding to calcium phosphate^13^ ^,^ ^16^ ^,^ ^26^. In our results, all materials showed ability to nucleate calcium phosphate on their surface. The Ca:P atomic ratio at 28 d was 2.05 for conventional MTA, 1.63 for MTA Repair HP and 1.87 for MTA Vitalcem. The lower Ca:P atomic ratio of MTA Repair HP could be correlated to the presence of the plasticizer. Although, further XDR analysis would precisely indicate the crystalline composition of the structures^3^.

## Conclusion

In summary, the study showed that the novel cements MTA Repair HP and MTA Vitalcem had extended alkalinizing activity and calcium release, which favored calcium phosphate nucleation. However, MTA Vitalcem showed scant radiopacity, a long setting time and high solubility in comparison with the other tested materials. MTA Repair HP showed similar results to MTA Angelus, but the presence of the plasticizer may have increased solubility and porosity. The radiopacifier calcium tungstate can be used to replace bismuth oxide.

## References

[1] Bortoluzzi EA, Broon NJ, Bramante CM, Garcia RB, Moraes IG, Bernardineli N (2006). Sealing ability of MTA and radiopaque Portland cement with or without calcium chloride for root-end filling. J Endod..

[2] Camilleri J, Gandolfi MG (2010). Evaluation of the radiopacity of calcium silicate cements containing different radiopacifiers. Int Endod J..

[3] Camilleri J, Montesin FE, Brady K, Sweeney R, Curtis RV, Ford TR (2005). The constitution of mineral trioxide aggregate. Dent Mater..

[4] Cintra LT, Benetti F, Azevedo Queiroz IO, Araújo Lopes JM, Penha de Oliveira SH, Sivieri Araújo G (2017). Cytotoxicity, biocompatibility, and biomineralization of the new high-plasticity MTA material. J Endod..

[5] Coomaraswamy KS, Lumley PJ, Hofmann MP (2007). Effect of bismuth oxide radioopacifier content on the material properties of an endodontic Portland cement-based (MTA-like) system. J Endod..

[6] Dawood AE, Manton DJ, Parashos P, Wong R, Palamara J, Stanton DP (2014). The physical properties and ion release of CPP-ACP-modified calcium silicate-based cements. Aust Dent J..

[7] Figueroa A, Obando G (2014). VITALCEM: a regenerational dental cement based on construction portland cement. Odontol Pediatr..

[8] Gandolfi MG, Iacono F, Agee K, Siboni F, Tay F, Pashley DH (2009). Setting time and expansion in different soaking media of experimental accelerated calcium-silicate cements and ProRoot MTA. Oral Surg Oral Med Oral Pathol Oral Radiol Endod..

[9] Gandolfi MG, Siboni F, Botero T, Bossù M, Riccitiello F, Prati C (2015). Calcium silicate and calcium hydroxide materials for pulp capping: biointeractivity, porosity, solubility and bioactivity of current formulations. J Appl Biomater Funct Mater..

[10] Gandolfi MG, Siboni F, Prati C (2012). Chemical-physical properties of TheraCal, a novel light-curable MTA-like material for pulp capping. Int Endod J..

[11] Gandolfi MG, Siboni F, Primus CM, Prati C (2014). Ion release, porosity, solubility, and bioactivity of MTA Plus tricalcium silicate. J Endod..

[12] Gandolfi MG, Spagnuolo G, Siboni F, Procino A, Rivieccio V, Pelliccioni GA (2015). Calcium silicate/calcium phosphate biphasic cements for vital pulp therapy: chemical-physical properties and human pulp cells response. Clin Oral Investig..

[13] Gandolfi MG, Taddei P, Modena E, Siboni F, Prati C (2013). Biointeractivity-related versus chemi/physisorption-related apatite precursor-forming ability of current root end filling materials. J Biomed Mater Res Part B Appl Biomater..

[14] Gandolfi MG, Taddei P, Siboni F, Modena E, Ciapetti G, Prati C (2011). Development of the foremost light-curable calcium-silicate MTA cement as root-end in oral surgery. Chemical-physical properties, bioactivity and biological behavior. Dent Mater..

[15] Gandolfi MG, Taddei P, Siboni F, Modena E, Ginebra MP, Prati C (2011). Fluoride-containing nanoporous calcium-silicate MTA cements for endodontics and oral surgery: early fluorapatite formation in a phosphate-containing solution. Int Endod J..

[16] Gandolfi MG, Taddei P, Tinti A, Dorigo ES, Prati C (2011). Alpha-TCP improves the apatite-formation ability of calcium-silicate hydraulic cement soaked in phosphate solutions. Mater Sci Eng C..

[17] Gandolfi MG, Van Landuyt K, Taddei P, Modena E, Van Meerbeek B, Prati C (2010). Environmental scanning electron microscopy connected with energy dispersive x-ray analysis and raman techniques to study ProRoot mineral trioxide aggregate and calcium silicate cements in wet conditions and in real time. J Endod..

[18] Hilton TJ, Ferracane JL, Mancl L (2013). Northwest Practice-based Research Collaborative in Evidence-based Dentistry (NWP). Comparison of CaOH with MTA for direct pulp capping: a PBRN randomized clinical trial. J Dent Res..

[19] Hungaro Duarte MA, Minotti PG, Rodrigues CT, Zapata RO, Bramante CM, Tanomaru M (2012). Effect of different radiopacifying agents on the physicochemical properties of white Portland cement and white mineral trioxide aggregate. J Endod..

[20] Húngaro Duarte MA, Oliveira El Kadre GD, Vivan RR, Guerreiro Tanomaru JM, Tanomaru M, Moraes IG (2009). Radiopacity of portland cement associated with different radiopacifying agents. J Endod..

[21] Jacinto RC, Linhares-Farina G, Sposito OS, Zanchi CH, Cenci MS (2015). Influence of 2% chlorhexidine on pH, calcium release and setting time of a resinous MTA-based root-end filling material. Braz Oral Res..

[22] Marciano MA, Costa RM, Camilleri J, Mondelli RF, Guimarães BM, Duarte MA (2014). Assessment of color stability of white mineral trioxide aggregate angelus and bismuth oxide in contact with tooth structure. J Endod..

[23] Margolis HC, Kwak SY, Yamazaki H (2014). Role of mineralization inhibitors in the regulation of hard tissue biomineralization: relevance to initial enamel formation and maturation. Front Physiol..

[24] Obando Pereda GA, Torres Chávez KE, Salas Beltrán H, Hofing JF (2009). Analysis of the chemical composition, apical sealing ability and antimicrobial properties of MTA and Portland cement. Endodoncia (Madrid)..

[25] Prati C, Gandolfi MG (2015). Calcium silicate bioactive cements: biological perspectives and clinical applications. Dent Mater..

[26] Silva EJ, Herrera DR, Rosa TP, Duque TM, Jacinto RC, Gomes BP (2014). Evaluation of cytotoxicity, antimicrobial activity and physicochemical properties of a calcium aluminate-based endodontic material. J Appl Oral Sci..

[27] Singh S, Podar R, Dadu S, Kulkarni G, Purba R (2015). Solubility of a new calcium silicate-based root-end filling material. J Conserv Dent..

[28] Souza LC, Yadlapati M, Dorn SO, Silva R, Letra A (2015). Analysis of radiopacity, pH and cytotoxicity of a new bioceramic material. J Appl Oral Sci..

[29] Vallés M, Mercadé M, Duran-Sindreu F, Bourdelande JL, Roig M (2013). Color stability of white mineral trioxide aggregate. Clin Oral Investig..

[30] Vallés M, Mercadé M, Duran-Sindreu F, Bourdelande JL, Roig M (2013). Influence of light and oxygen on the color stability of five calcium silicate-based materials. J Endod..

[31] Vivan RR, Zapata RO, Zeferino MA, Bramante CM, Bernardineli N, Garcia RB (2010). Evaluation of the physical and chemical properties of two commercial and three experimental root-end filling materials. Oral Surg Oral Med Oral Pathol Oral Radiol Endod..

